# Acute Case of Trichobezoar Diagnosed From Computed Tomography and 3D Images: Rapunzel Syndrome Re-examined

**DOI:** 10.7759/cureus.35597

**Published:** 2023-02-28

**Authors:** Cynthia Kyin, Pushpak Patel, Adela Casas-Melley, Wael M Abdalla, Tamarah Westmoreland

**Affiliations:** 1 Radiology, University of Central Florida College of Medicine, Orlando, USA; 2 3D Printing Lab, Nemours Children's Hospital, Orlando, USA; 3 Pediatric Surgery, Nemours Children's Hospital, Orlando, USA; 4 Radiology, Nemours Children's Hospital, Orlando, USA; 5 Pediatric Surgery, University of Central Florida College of Medicine, Orlando, USA

**Keywords:** trichotillomania, pediatric lap. surgery, gastric bezoar, 3d ct scan, rapunzel syndrome

## Abstract

A trichobezoar is a rare cause of abdominal pain due to an indigestible mass in the gastrointestinal tract that is composed of a patient’s hair. If a trichobezoar grows and extends from the gastric body to the pylorus and into the small bowel, it is considered Rapunzel syndrome. We present a case of an 11-year-old female patient with Rapunzel syndrome who presented with four weeks of colicky abdominal pain, vomiting, constipation, and severe malnutrition. Computed tomography of the abdomen and pelvis with 3D rendering demonstrated a large bezoar, and the patient was successfully treated with exploratory laparotomy, gastrostomy, and removal of the trichobezoar intact.

## Introduction

A bezoar is a mass of undigested material in the gastrointestinal tract [1,2]. Bezoars can be further categorized based on their contents. Phytobezoars are comprised of indigestible vegetables and fruits, trichobezoars are primarily made up of a patient’s hair and may include hair from carpets or animals, pharmacobezoars consist of medication, and other bezoars are composed of miscellaneous materials such as paper, styrofoam, or gloves [2,3]. A relatively uncommon form of a trichobezoar is Rapunzel syndrome where the bezoar extends from the gastric body through the pylorus and into the small bowel [4,5]. 

Rapunzel syndrome was initially reported in 1968 by Vaughan et al. and is primarily seen in young females [4,6-8]. Trichophagia, i.e., the swallowing of one’s hair is seen in all of these patients while trichotillomania, i.e., the urge to pull out one’s own hair is also seen in many patients with this disorder [9,10]. Trichobezoars may present with a vast array of symptoms such as abdominal pain, anorexia, obstruction, and peritonitis [11,12]. Given its unspecific symptoms, trichobezoars may go undiagnosed for years [9]. If unrecognized, trichobezoars will continue to grow and can potentially cause complications such as gastric mucosal erosions, ulcerations, or possibly even perforation [7,13].

We describe a case of an 11-year-old girl who presented with a progressive obstruction from a trichobezoar that occupied her stomach and first part of the duodenum with associated Rapunzel syndrome.

## Case presentation

An 11-year-old female presented to her primary care doctor with a four-week history of colicky periumbilical abdominal pain, daily vomiting, constipation, and weight loss. The abdominal pain did not follow a specific pattern and was sometimes postprandial while also sometimes during the middle of the night. The patient also had severe malnutrition based on a 4 kg weight loss over the past month, which was 12.5% of her baseline weight. The patient’s symptoms were initially mild and were first noted at a well-child visit a month prior to admission, and she was advised to try a lactose-free diet. However, her symptoms continued to progress, and she was seen in the emergency department on two different occasions prior to being admitted. During the first emergency department visit, she presented with abdominal pain, fever, nausea, and vomiting, was diagnosed with gastroenteritis, and was discharged on ondansetron. On the second emergency visit, the patient was diagnosed with constipation from abdominal x-ray and was discharged on polyethylene glycol. Her symptoms continued to persist despite increasing her polyethylene glycol. 

Her past medical history is significant for a long history of eating her hair as well as chewing on blankets. However, she had no formal diagnosis of trichophagia or pica. The patient’s parents also note that the child is sometimes anxious; however, she has not had a formal diagnosis of this either. The patient had no history of previous abdominal surgery. Vitals were significant for a pulse of 120 and a respiratory rate of 24. Physical exam revealed normoactive bowel sounds and a softly distended abdomen with a firm mass in the epigastric region. Given the progression of her symptoms, the patient was admitted for further workup for her abdominal pain and vomiting.

**Figure 1:**
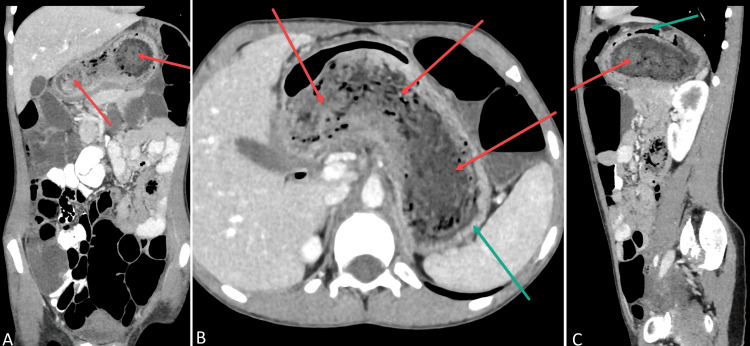
Computed tomography images - coronal (A), axial (B) and sagittal (C) views of abdomen showing stomach bezoar (red arrows) extending through entire stomach (green arrows).

**Figure 2:**
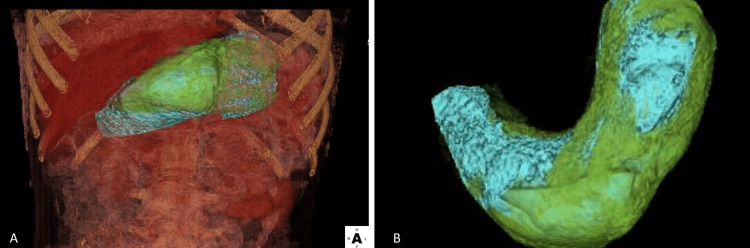
(A) 3D rendered image of surrounding organs and intestine showing stomach bezoar (blue) in the stomach (green) (B) isolated 3D rendered image of stomach bezoar (blue) within the stomach wall (green)

Computed tomography imaging of the abdomen and pelvis (CTAP) revealed a heterogenous density, not attached to the stomach wall that did not show enhancement and was consistent with a large bezoar (Figure 1). The mass extended from the gastric lumen to the first part of the duodenum. Additionally, the stomach wall demonstrated mild wall thickening with mucosal enhancement suggesting reactive gastritis. There were no signs of bowel obstruction. 3D rendering of the mass on CTAP allowed for better visualization of the mass and can be seen in Figure 2. Given the characteristic findings of a bezoar on CTAP, an endoscopy was not performed because this would not adequately remove the bezoar mass.

**Figure 3:**
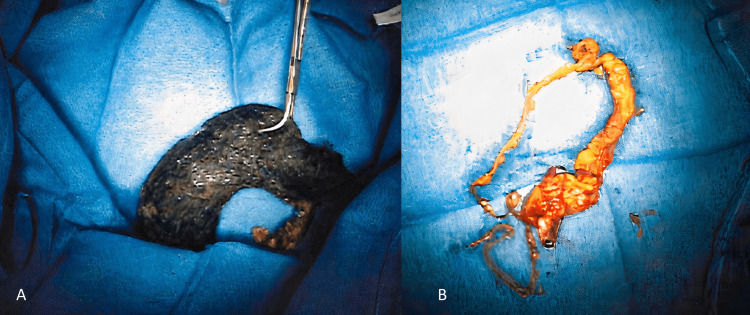
(A) Large gastric trichobezoar removed from the stomach. (B) Second fragment of the trichobezoar which extended through the pylorus.

The patient underwent an exploratory laparotomy the next day with an upper midline incision. The stomach was exposed, and the large bezoar was palpated. The body of the stomach was opened with a six cm horizontal incision, and the gastric trichobezoar was removed intact (Figure 3A). Additionally, hair extending through the pylorus was extracted as well by grasping the hair and pulling it from the duodenum. On gross examination, the bezoar was assessed as two fragments with the larger segment measuring 12 x 9 x 5.5 cm in size and primarily comprising hair. The second fragment was 6.5 x 4.5 x 3 cm in size and appeared to be made up of hair and brown-tan material as well (Figure 3B).

The patient’s recovery from surgery was uneventful, and she was discharged three days following surgery. Pain was controlled with ketorolac, acetaminophen, and morphine as needed. Patient was advanced to a regular diet on postoperative day three. The patient was seen by an inpatient psychologist who diagnosed her with pica and adjustment disorder with anxious mood. Outpatient cognitive behavioral psychotherapy was also recommended to improve coping strategies and decrease symptoms of stress.

## Discussion

One of the most common chief complaints in the pediatric population is abdominal pain [13]. An exceptionally rare etiology of abdominal pain is trichobezoars with an incidence rate of about less than 1% [11]. Most commonly, trichobezoars present in young female patients with coexisting psychiatric disorders [6]. An even more rare manifestation of a trichobezoar is Rapunzel syndrome where the bezoar extends past the pylorus and into the duodenum. Given the low incidence of Rapunzel syndrome, it can often go undiagnosed and may require multiple investigations [14]. Furthermore, without a knowledge of this disorder, the portion of hair beyond the pylorus could be missed and result in continued symptoms and repeat operation. Imaging is a key component in diagnosing this disorder preoperatively. As seen in this case, a detailed history as well as high suspicion is necessary to diagnose a trichobezoar.

Often patients with bezoars remain asymptomatic for long periods of time and tend to not present until after the bezoar has grown, which can result in complications. The most common symptoms of a bezoar are abdominal pain, vomiting, abdominal distention, and weight loss. The current patient presented with all of these symptoms. Although rare, reported complications of Rapunzel syndrome include perforation and pancreatitis due to the bezoar obstructing the ampulla of Vater [14]. Given the severity of the possible complications, bezoars should be included in the differential when assessing patients with chronic nonspecific abdominal pain, especially in those with a history of trichophagia.

Initial workup for abdominal pain can include the use of plain radiographs and ultrasound. Plain radiographs are often sensitive enough to detect a bowel obstruction; however, radiologists are rarely able to use radiographs alone to identify bezoars as the etiology of a bowel obstruction with two previous studies reporting identification rates of 10% and 18% [15,16]. The sensitivity of ultrasound for detecting bezoars is 88%, and they can be characterized as a hyperechoic intraluminal mass with marked acoustic shadowing [17]. However, ultrasound has decreased sensitivity when detecting gastric bezoars that are associated with intestinal bezoars with a previous study reporting an identification rate of 25% [15]. Magnetic resonance imaging (MRI) is also a potential option for diagnosing a bezoar. However, the appearance of a bezoar changes depending on the amount of air, water, fat, or food within the bezoar. Additionally, since bezoars can be confused with air on MRI due to their signal characteristics, MRI is a less useful technique given that they are also time-consuming and expensive [18].

In contrast to the previously stated imaging techniques, CTAP has been reported to be highly effective in identifying bezoars with an identification rate of 97% with the added benefit of establishing the size, location, extent of obstruction as well as ruling out other possible etiologies for the abdominal pain [6]. Another advantage of the CTAP is the ability to use 3D rendering to further assess and characterize the bezoar. As seen in the current patient, the 3D visualization of the bezoar allowed for increased certainty in the diagnosis and improved presurgical planning. Given the added information of CTAP with 3D rendering, this case highlights the value that this technology adds in assessing patients with suspected bezoars and decreasing the risk of a repeat operation for a missed portion of the bezoar.

Following diagnosis, treatment for trichobezoars varies depending on the location and size. Smaller trichobezoars may be removed using endoscopy while exploratory laparotomy is the preferred approach for larger bezoars [14]. Rapunzel syndrome in itself is an indication for exploratory laparotomy since it permits proper examination of the gastrointestinal tract and allows for proper management if there are complications [1]. The success rate of this approach is close to 100% relative to 75% and 5% for laparoscopy and endoscopy, respectively [1,19]. In our case, the patient underwent successful laparotomy with complete removal of the trichobezoar.

As a result of the low incidence of trichobezoars, there is little consensus on post-operative follow-up guidelines to prevent recurrence. However, previous studies have suggested biannual abdominal imaging as well as routine ultrasound or upper gastrointestinal endoscopy at the six-, 12-, and 24-month follow-up periods [20]. In a patient with a history of trichobezoars, symptoms such as weight loss, anemia, or parental concern should prompt early endoscopic evaluation [1]. Further, adequate behavioral therapy for controlling trichophagia and psychological support is imperative for avoiding recurrence.

## Conclusions

Trichobezoars and specifically Rapunzel syndrome are a rare etiology of abdominal pain. Patients may present with vague symptoms, so high suspicion is necessary to quickly diagnose these patients. CTAP is currently the most effective imaging modality for diagnosis, and 3D-rendered images can be used to further characterize and assess the mass. First-line treatment for Rapunzel syndrome is exploratory laparotomy as well as multidisciplinary care that includes routine imaging at follow-up appointments and psychological support to prevent recurrence.
